# Paraneoplastic Aquagenic Pruritus: A Case of Pancreatic Cancer

**DOI:** 10.7759/cureus.35566

**Published:** 2023-02-28

**Authors:** Catarina Negrão, Marta Machado, Margarida Mourato, Rita Sismeiro, Marta Jonet

**Affiliations:** 1 Internal Medicine, Hospital Professor Doutor Fernando Fonseca, Amadora, PRT

**Keywords:** pancreatic cancer, deep vein thrombosis, aquagenic pruritus, paraneoplastic pruritus, paraneoplastic syndrome

## Abstract

Paraneoplastic pruritus has been reported mostly in association with haematological malignancies, and rarely with solid tumours. Aquagenic pruritus is itching without any skin lesion that develops a few minutes after contact with water of any temperature and it is associated with polycythaemia vera or other lymphoproliferative diseases.

Here we report a case of a previously healthy 78-year-old Portuguese woman, who had been treated unsuccessfully for aquagenic pruritus for the previous eight months, and presented to the emergency department complaining of pain and swelling in her left leg. Deep vein thrombosis was diagnosed and oral anticoagulation was initiated. Blood tests revealed a normal blood count and normal liver enzymes, except for alkaline phosphatase and lactate dehydrogenase levels, which were slightly elevated. Hypercobalaminaemia and folic acid deficiency were also noted. JAK2 V617F/12 exon mutation was not present. Thoracic, abdominal and pelvic computed tomography revealed a locally advanced pancreatic tumour. Ultrasound-guided fine-needle aspiration cytology of the lesion revealed a moderately differentiated adenocarcinoma of pancreatic ductal origin. Tumour marker assays showed elevation of both carcinoembryonic antigen (CEA) and carbohydrate antigen 19-9 (CA 19-9).

Aquagenic pruritus should be thoroughly investigated to exclude a neoplastic disease, especially if treatment is refractory or if another paraneoplastic syndrome is present. Although aquagenic pruritus is more commonly associated with haematological malignancies than solid tumours, a rare case of aquagenic pruritus is described here as a paraneoplastic syndrome of pancreatic cancer. To the best of our knowledge, this is the first case of pancreatic cancer that presented with aquagenic pruritus and dual paraneoplastic syndromes.

## Introduction

Pancreatic cancer is often a “silent” disease, commonly asymptomatic at early stages. When symptoms do occur, they are often vague and vary depending on the location of the tumour. In most cases, pancreatic tumours cause clinical symptoms because of local expansion, compression and infiltration of blood vessels, lymph vessels, nerve fibres or surrounding organs. Therefore, abdominal pain and jaundice are among the most important clinical signs of pancreatic cancer. Although rare, patients may also report pruritus secondary to a mechanical obstruction of the bile duct, causing cholestatic pruritus, or it may be secondary to a paraneoplastic syndrome causing paraneoplastic pruritus.

Paraneoplastic syndromes are defined as signs and symptoms that present distantly from the primary cancer or site of metastases, with multiple clinical symptoms, including systemic and organ-specific manifestations [1]. The precise incidence and prevalence of paraneoplastic syndrome are unknown due to the rarity of the disease. A literature review suggests that paraneoplastic syndrome occurs in up to 8% of cancer patients. Paraneoplastic pruritus is defined as the sensation of itch as a systemic (not local) reaction to the presence of a tumour or an haematological malignancy that is not triggered by the local presence of cancer cells or by tumour therapy. It usually disappears with the remission of the tumour and may recur if there is a relapse. It can occur as a single symptom or with different clinical and pathophysiological signs [2]. Aquagenic pruritus is pruritus without any skin lesions that develops minutes after contact with water of any temperature. Paraneoplastic aquagenic pruritus is commonly associated with polycythemia vera (PV) or other lymphoproliferative diseases, but rarely with solid tumours [3].

We present a case of paraneoplastic aquagenic pruritus in a patient with locally advanced pancreatic adenocarcinoma. To our knowledge, and after reviewing the literature, this is not only the first case of paraneoplastic aquagenic pruritus associated with pancreatic adenocarcinoma, but also the first case of dual paraneoplastic syndromes preceding the diagnosis of a pancreatic adenocarcinoma.

This article was previously presented as a meeting abstract at the 2022 Congresso Nacional de Medicina Interna on October 4, 2022.

## Case presentation

A 78-year-old Portuguese woman with no known medical conditions presented to the emergency department complaining of pain and swelling in her left leg. She used to be a fully active woman who was not taking any medication. She was an active smoker (11 packs per-year) and there was no personal or family history of malignancy. She had not had any recent surgery and her last trip (and associated immobilisation) had been one month earlier, to visit her daughters in England.

On physical examination, the patient was found to have tenderness in the left calf, with no palpable cords. There was pain when flexing her left calf muscle with plantar flexion of the foot. Her vital signs were unremarkable, with no tachycardia or respiratory distress. No routine laboratory tests were performed at the first hospital visit and an ultrasound scan of the lower extremity revealed iliofemoral deep vein thrombosis. She was discharged with a prescription of an oral anticoagulant and referred to an Internal Medicine consultation.

At her first appointment, she denied weight loss, fever, night sweats, postprandial fullness, new gastrointestinal symptoms or abnormal bleeding. Her only complaint was the presence of aquagenic pruritus for the past eight months, for which she was consulted at a private clinic and subsequently treated with emollients, H1-receptor antagonist antihistamines and H2-receptor antagonists (all at maximum dosage), which did not provide adequate relief.

A mammogram performed five years earlier was benign, with a score of 2 according to the Breast Imaging Reporting and Data System (BI-RADS). Her complete blood count was normal, with no anaemia (hemoglobin 13 g/dL; reference range 12.0-15.0 g/dL), leukocytosis (7.2x10^9^/L; reference range 4.0-10.0x10^9^/L) or thrombocytosis (platelet count 235x10^9^/L; reference range 150-410x10^9^/L). Liver enzymes, including γ-glutamyl transferase, alanine aminotransaminase, aspartate aminotransferase and total bilirubin, were within the normal range; however, alkaline phosphatase (reference range 35-105 IU/L) and lactate dehydrogenase (reference range 135-214 IU/L) were slightly elevated (150 and 249 IU/L, respectively). A high serum cobalamin level (1196 pg/mL; reference range 200-900 pg/mL) and folate deficiency (2.9 ng/mL; reference range 4-20 ng/mL) were noted. All main laboratory findings are presented in Table 1.

**Table 1:** Laboratory investigation results

Laboratory study	Result	Reference range
Hemoglobin (g/dL)	13	12-15
White blood cell count (/µL)	7,200	4,000-10,000
Platelet count (/µL)	235,000	150,000-410,000
Alanine aminotransaminase (IU/L)	23	<32
Aspartate aminotransferase (IU/L)	26	<33
Alkaline phosphatase (IU/L)	150	35-105
Gamma-glutamyl transferase (IU/L)	38	<40
Total bilirubin (mg/dL)	0.21	<1.20
Lactate dehydrogenase (IU/L)	249	135-214
Serum cobalamin (pg/mL)	1196	200-900
Serum folate (ng/mL)	2.9	4-20

**Figure 1:**
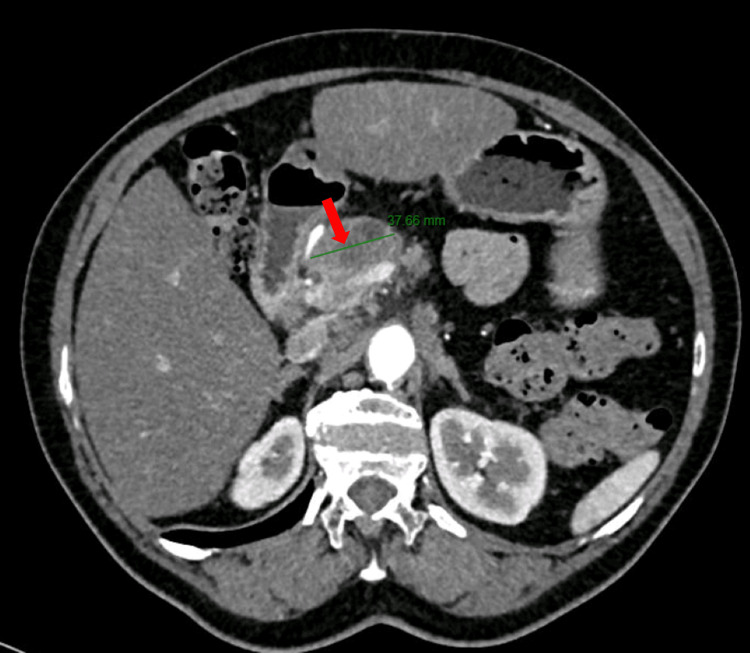
Abdominal computed tomography showing a 3.7 cm (greater axis) infiltrative lesion of the head of the pancreas (red arrow)

Due to aquagenic pruritus and to rule out PV, genetic testing for the JAK2 V617F/Exon 12 mutation was performed and was found out to be negative. Considering the possibility of facing two paraneoplastic syndromes, i.e. spontaneous deep vein thrombosis and aquagenic pruritus, thoracic, abdominal and pelvic computed tomography (CT) was performed, which showed a 3.7 cm (greater axis) infiltrative lesion of the pancreatic head, causing dilatation of the Wirsung duct in the body and tail of the pancreas (Figure 1).

**Figure 2:**
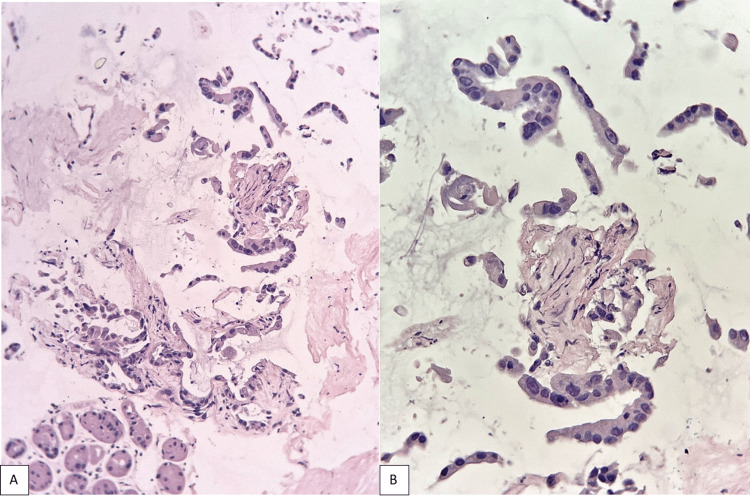
Pancreatic ductal adenocarcinoma Cell block, hematoxylin and eosin stain, 100x (A) and 400x (B)

This mass involved the spleno/portal venous confluence, the common hepatic artery, the celiac trunk and the splenic artery. Hepatic hilum and retroperitoneal adenomegalies were also described. Ultrasound-guided fine-needle aspiration cytology of the lesion was obtained, which reported moderately differentiated adenocarcinoma of pancreatic ductal origin (Figure 2).

According to the International Union Against Cancer TNM classification (eighth edition), the conclusive stage of pancreatic cancer was T4N2M0, stage III [4]. Tumour marker assays were elevated, with a carcinoembryonic antigen (CEA) level of 8.3 ng/mL (reference range is <2.5 ng/mL in an adult non-smoker and <5.0 ng/mL in a smoker) and a carbohydrate antigen 19-9 (CA 19-9) level of 842 IU/mL (reference range 0-37 IU/mL).

The case was presented and discussed at the multidisciplinary tumour board, and systematic palliative chemotherapy was planned, which the patient started in April 2022 (one month after the first Internal Medicine appointment). The patient was referred to an oncologist and a good response was noted after starting treatment: a decrease in tumour marker assays was reported at each monthly evaluation and after five months of treatment, the patient reported that the aquagenic pruritus had finally disappeared.

## Discussion

Pancreatic cancer is known as an aggressive disease with a poor prognosis, accounting for almost as many deaths as new cases yearly. The total number of deaths due to pancreatic cancer is projected to increase dramatically and become the second leading cause of cancer-related deaths by 2030 [5]. Risk factors are not easy to identify. There are hereditary risk factors (10%), such as genetic pattern, and environmental (modifiable) factors such as smoking, alcohol consumption, chronic pancreatitis, obesity and diabetes mellitus [6]. The risk of developing pancreatic cancer increases with age: about 80% patients are at least 60 years old and 71 years is the average age at diagnosis [7].

Pancreatic cancer is known as the “silent killer” because its symptoms are vague and non-specific, leading to a difficult and often late diagnosis, usually at an advanced stage. Sometimes the first sign is a paraneoplastic syndrome, which may precede the more classic symptoms of pancreatic cancer. Clinical symptoms vary widely and include systemic (such as fever and cachexia) and organ-specific manifestations, such as cutaneous, neurological, haematological (most commonly anaemia, leukocytosis and thrombocytosis), or endocrine symptoms [1]. Pancreatic cancer remains one of the most prothrombotic neoplasms, with an incidence of thrombotic disease of up to 36% [8]. Consequently, venous thrombosis and pulmonary embolism are quite common in this type of cancer. Although rare, patients can present with multiple paraneoplastic syndromes simultaneously, especially if the tumour originates from neuroendocrine cells [9]. In the literature, there have been patients with two or more paraneoplastic syndromes; however, to the best of our knowledge, the association of dual paraneoplastic syndromes and pancreatic cancer has not yet been reported.

Aquagenic pruritus is a subtype of paraneoplastic itch. It is characterised by itching, sometimes accompanied by a burning or stinging sensation triggered by contact with water, regardless of the temperature and without any cutaneous changes. Symptoms may appear immediately after contact with water and last for an hour or more. Most cases are idiopathic and related only to the water itself. However, it is often associated with PV, in which a JAK2 mutation is detected in up to 99% of patients [10]. Aquagenic pruritus may precede the development of PV by several years [3]. Several other reports have demonstrated an association with conditions such as juvenile xanthogranuloma, myelodysplastic syndrome, acute leucocytoclastic vasculitis, lymphoblastic leukaemia, T-cell non Hodgkins's lymphoma, metastatic squamous cell carcinoma, hepatitis C infection, idiopathic hypereosinophilic syndrome, and with drugs like bupropion and antimalarials in cumulative doses, when used to treat systemic lupus erythematosus [11-20].

Because aquagenic pruritus may be associated with many diseases, especially PV and other tumours whose prognosis depends on prompt diagnosis and initiation of therapy, a high degree of suspicion is essential in a patient with aquagenic pruritus who either does not respond adequately to first-line treatment or presents with another symptom that may be associated with a paraneoplastic syndrome. In this case, only after an adequate anamnesis and a complete objective examination, was aquagenic pruritus valued as part of the problem.

In this case, in view of two possible paraneoplastic syndromes, aquagenic pruritus and deep vein thrombosis, the urge to rule out an occult malignancy (namely PV or another neoplastic lesion) prompted us to investigate with body CT and a genetic test for the JAK2 V617F/Exon 12 mutation. The diagnosis of pancreatic adenocarcinoma was quickly made and chemotherapy was immediately started, after which the blood values (tumour marker assays) and symptoms improved. Although there are many different aetiologies for aquagenic pruritus, after a thorough review of the literature we found no evidence of aquagenic pruritus as the first manifestation of pancreatic cancer, making this case the first reported.

## Conclusions

In the presence of a symptom possibly suggestive of a paraneoplastic syndrome, a complete anamnesis and objective examination are essential. The presence of more than one paraneoplastic syndrome should definitely alert the physician to a possible occult malignancy, even in the absence of systemic or local manifestations, such as weight loss, fever, anorexia or abdominal pain. Although aquagenic pruritus is more commonly associated with haematological malignancies, in their absence a thorough review of patient records should be performed to ensure the exclusion of an underlying malignancy, as described in this case.
